# Phosphorylation-Dependent Regulation of WNT/Beta-Catenin Signaling

**DOI:** 10.3389/fonc.2022.858782

**Published:** 2022-03-14

**Authors:** Kinjal Shah, Julhash U. Kazi

**Affiliations:** ^1^ Division of Translational Cancer Research, Department of Laboratory Medicine, Lund University, Lund, Sweden; ^2^ Lund Stem Cell Center, Department of Laboratory Medicine, Lund University, Lund, Sweden

**Keywords:** β-catenin, *CTNNB1*, GSK3β, adherens junctions, AXIN, CK1, frizzled

## Abstract

WNT/β-catenin signaling is a highly complex pathway that plays diverse roles in various cellular processes. While WNT ligands usually signal through their dedicated Frizzled receptors, the decision to signal in a β-catenin-dependent or -independent manner rests upon the type of co-receptors used. Canonical WNT signaling is β-catenin-dependent, whereas non-canonical WNT signaling is β-catenin-independent according to the classical definition. This still holds true, albeit with some added complexity, as both the pathways seem to cross-talk with intertwined networks that involve the use of different ligands, receptors, and co-receptors. β-catenin can be directly phosphorylated by various kinases governing its participation in either canonical or non-canonical pathways. Moreover, the co-activators that associate with β-catenin determine the output of the pathway in terms of induction of genes promoting proliferation or differentiation. In this review, we provide an overview of how protein phosphorylation controls WNT/β-catenin signaling, particularly in human cancer.

## Introduction

WNT/β-catenin signaling is a tightly controlled and highly conserved pathway that regulates cell fate during embryogenesis, hepatobiliary development, liver homeostasis, repair in adulthood, cell proliferation, differentiation, and cell polarity (1, 2). The intracellular responses that WNT ligands trigger can be classified into canonical (β-catenin-dependent) and non-canonical (β-catenin-independent) signaling (2, 3). WNT is a family of nineteen hydrophobic cysteine-rich secreted glycoproteins which serve as ligands for ten members of the Frizzled (Fz) family of 7-transmembrane receptors, the co-receptors low-density lipoprotein receptor-related proteins 5/6 (LRP 5/6), and non-classical WNT receptors like RYK and ROR (4–8). Abnormal WNT/β-catenin signaling is involved in many diseases including Alzheimer’s disease, heart disease, osteoarthritis, and cancer (9, 10). β-catenin is one of the core molecules in the canonical WNT signaling pathway. It is also involved in E-cadherin and cytoskeleton-associated cell-cell adhesion when localized to the plasma membrane (11, 12). However, cytosolic β-catenin acts as the molecular effector of the WNT ligands (1, 13). This review discusses the phosphorylation-dependent regulations of β-catenin and WNT signaling in cancer.

## Structure, Location, and Function of β-Catenin

**Figure 1:**
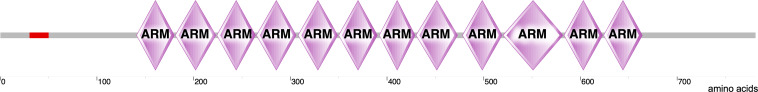
Structure of β-catenin. The structure of β-catenin was generated by SMART (http://smart.embl-heidelberg.de/smart/show_motifs.pl?ID=P35222) and modified using Canvas X draw. It includes an N-terminal domain where several regulatory phosphorylation sites are located (red). This domain follows twelve ARM domains and a long C-terminal domain.

β-catenin is a multifunctional protein encoded by the *CTNNB1* gene in humans and is the vertebrate homolog of the Drosophila Armadillo (14). It is a 781-amino-acid-long protein consisting of the N-terminal domain (NTD), twelve armadillo (ARM) domains in the middle of the protein, and the C-terminal domain (CTD) (**Figure 1**). Each ARM domain contains three α-helices and, together, all twelve ARM domains create a compact superhelix with a positively charged groove spanning all the ARM domains (15, 16). This core ARM domain structure serves as a scaffold and interacts with various β-catenin binding partners that are critical for both WNT signaling and the formation of adherens junctions (16, 17). β-catenin can exist in three distinct pools inside the cell: membranous, cytoplasmic, and nuclear (18). β-catenin normally interacts with E-cadherin at the cell membrane and plays an important structural role in the adherens junctions. β-catenin that is free in the cytoplasm is captured by the destruction complex for degradation. However, when some of the components of the destruction complex are compromised, β-catenin evades degradation; instead, it translocates to the nucleus and contributes to the transcriptional regulation of genes (18). β-catenin thus acts as both an adaptor protein and a transcriptional coregulator (19). This spatial separation of β-catenin at the plasma membrane, cytoplasm, and the nucleus is regulated by specific phosphorylation mechanisms.

## Stabilization of β-Catenin at the Plasma Membrane as an Intracellular Adhesion Regulator

β-catenin acts as an adaptor protein and binds to the intracellular part of E-cadherin present at the plasma membrane *via* its C-terminal region. Apart from β-catenin, the cytoplasmic tail of E-cadherin can interact with various molecules such as γ-catenin and other regulatory proteins, while its extracellular part interacts with other cadherins present on adjacent cells (20, 21). The N-terminus of β-catenin interacts with α-catenin, which links β-catenin to the actin cytoskeleton. This entire structure of actin filaments-α-catenin-β-catenin-E-cadherin interactions promote clustering of the adhesion junction proteins, thereby stabilizing cell adhesion (22). In the absence of WNT, the majority of β-catenin localizes to the plasma membrane, building an epithelial barrier and restricting cell invasion and metastasis (23). However, the β-catenin-E-cadherin complex gets weakened by the phosphorylation of β-catenin at the plasma membrane (24). Tyrosine phosphorylation in the different armadillo repeats probably specifies interactions with α-catenin and E-cadherin. Phosphorylation of Tyr142/Tyr654 results in the dissociation of the adherens junctions, which leads to the cytoplasmic accumulation of β-catenin followed by its nuclear translocation to promote gene transcription (25). 

## Transcriptional Regulations Through Stabilization of β-Catenin in the Cytoplasm and Nucleus

**Figure 2:**
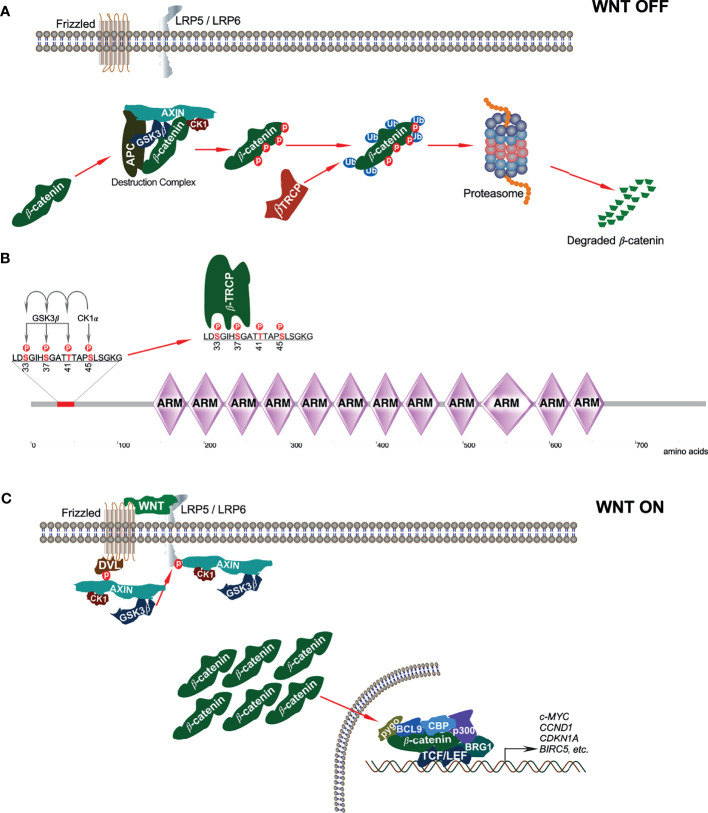
Canonical WNT signaling. **(A)** In absence of canonical WNT ligands, β-catenin is associated with the destruction complex. This interaction leads to phosphorylation-dependent ubiquitination of β-catenin and thereby its degradation in the proteasome. **(B)** In the destruction complex, CK1α phosphorylates β-catenin on Ser45 residue that initiates sequential phosphorylation of Thr41, Ser37, and Ser33 phosphorylation by GSK3β. Ser33 and Ser37 phosphorylation sites facilitate β-TRCP interaction with β-catenin. **(C)** In the presence of classical WNT ligands, a destruction complex cannot be formed and thus, β-catenin is stabilized from degradation.

In the absence of WNT signaling, β-catenin is sequestered in the cytoplasm by the ‘destruction complex’ composed of the scaffolding protein AXIN, tumor suppressor adenomatous polyposis coli (APC), and two serine/threonine kinases: casein kinase 1 (CK1) and glycogen synthase kinase 3β (GSK3β) (**Figure 2A**). AXIN directly binds with APC, GSK3β, CK1, and β-catenin and holds the destruction complex (26–29). Besides interaction with kinases, AXIN has an interaction site for protein phosphatase 2A (PP2A) that induces dephosphorylation of AXIN (30). Thus, AXIN is the core protein of the destruction complex that mediates the whole assembly of the destruction complex. Although AXIN can hold all proteins, the interaction between APC and β-catenin is required for active complex formation (31). GSK3β phosphorylates both AXIN (32, 33) and APC, which further increases its β-catenin binding affinity (34). CK1α binds to the first ARM domain of β-catenin (35) and mediates the first regulatory phosphorylation at Ser45 (36); this process requires AXIN-CK1α complex formation (29). Additionally, α-catenin must be dissociated from β-catenin to provide CK1 access. This dissociation is achieved by Tyr142 phosphorylation, which is mediated by tyrosine kinases FEline Sarcoma (FES)-related (FER) and FYN (35, 37). FYN is a member of SRC family of protein tyrosine kinases (SFKs). CK1α-induced Ser45 phosphorylation creates a priming site for GSK3β, which is necessary and sufficient for GSK3β-mediated phosphorylation at Thr41, Ser37, and Ser33 (29, 36). Ser33 and Ser37 in β-catenin create docking sites for the E3 ubiquitin ligase, β-transducing repeat-containing protein (β-TRCP) (**Figure 2B**) that ubiquitinates β-catenin and targets it for proteasomal degradation after forming a complex with Skp1 and Cullin (24, 38). Mutation of Ser37 thus results in stabilization of β-catenin (39).

The destruction complex is ultimately responsible for the phosphorylation of β-catenin, thereby priming it for ubiquitin-mediated proteasomal degradation (31). However, when canonical WNT ligands bind to their respective Fz receptor and LRP5/6 co-receptors, the result is the activation of the canonical WNT signaling pathway (40). Due to this, the phosphoprotein dishevelled (DVL, DVL1/2/3, segment polarity protein) is activated and recruited to the plasma membrane where it interacts with the cytoplasmic domain of Fz (41). DVL then recruits the destruction complex to the plasma membrane, thereby promoting interaction between LRP5/6 and AXIN (42, 43). GSK3β and CDK14 facilitate LRP5/6 phosphorylation that further enables AXIN-CK1-GSK3β complex recruitment (9). Recruitment of this complex to WNT receptors disrupts it and thereby inhibits CK1-GSK3β-mediated β-catenin phosphorylation, resulting in the stabilization and accumulation of β-catenin in the cytoplasm (**Figure 2C**).

Inhibition of CK1, WNT signaling activation, and DVL overexpression suppresses Ser45 phosphorylation (29). Moreover, GSK3β can be inactivated through Ser9 phosphorylation by AKT. These events result in the stabilization and accumulation of β-catenin in the cytoplasm (44). β-catenin activity in the canonical WNT pathway can also be regulated in a GSK3β and β-TrCP-independent manner.

It was observed in Drosophila that upon canonical WNT signaling, Arrow (LRP5/6) recruits AXIN to the membrane, which leads to degradation of AXIN. Thus, the scaffolding member of the destruction complex, AXIN, is no longer present to form the destruction complex (45). A more recent study demonstrated that an E3 ubiquitin ligase, tripartite motif-containing protein 11 (TRIM11), serves as an oncogene in lymphomas by promoting cell proliferation through activation of the β-catenin signaling. This regulation is brought about by TRIM11-mediated ubiquitination and degradation of AXIN1, part of the destruction complex of β-catenin (46). Thus, inactivation or degradation of any of the components forming the β-catenin destruction complex results in the stabilization and accumulation of β-catenin in the cytoplasm.

Stabilized β-catenin then translocates to the nucleus, where it interacts with different transcription factors, notably T-cell factor (TCF), and lymphoid enhancing factor (LEF). The repressor of the TCF/LEF complex, Groucho, is displaced upon this interaction, whose function is to compact chromatin (44). Thereafter, transcriptional co-activators and histone modifiers are recruited, which is sometimes referred to as WNT enhanceosome. These include the cyclic adenosine mono-phosphate response element (CREB)-binding protein (CBP), its closely related homolog p300, B-cell lymphoma 9 (BCL9), pygopus, and ATP-dependent helicase Brahma-related gene 1 (BRG1, also known as SMARCA4) (44, 47). Chromatin is remodeled by the WNT enhanceosome and results in the transcription of WNT/β-catenin target genes that are involved in cell survival and growth such as *c-MYC*, *CCND1*, *CDKN1A*, and *BIRC5* (40). *C-MYC* is a proto-oncogene that further activates cyclin D1 and also inhibits the tumor suppressors p21 and p27, thereby leading to uncontrolled cell proliferation (48, 49).

## Mutations of Components Involved in the Canonical WNT/β-Catenin Pathway

**Figure 3:**
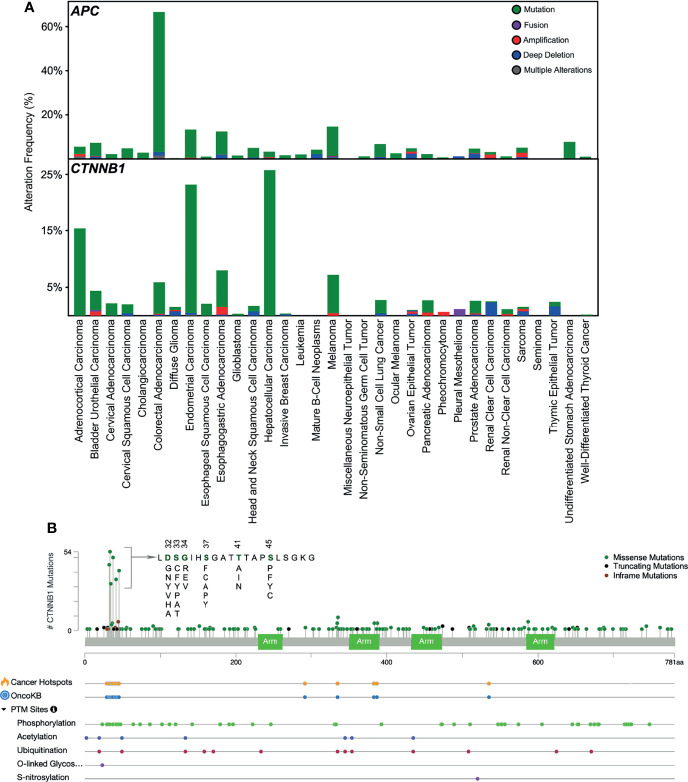
Mutations in APC and β-catenin. **(A)** Mutations in *APC* and *CTNNB1* (β-catenin) in different cancers have been collected from cbioportal. **(B)** Mutation frequency in *CTNNB1* gene and other post-translational modifications collected from cbioportal. TCGA PanCancer Atlas Studies (http://www.cbioportal.org/study/summary?id=5c8a7d55e4b046111fee2296) was used.

After having understood the regulation of β-catenin in the canonical WNT pathway, we now know that mutations in any of the components of this pathway can lead to its deregulation, which can contribute to a variety of diseases. Herein, we will focus on such mutations contributing to cancer. APC plays an important role in β-catenin degradation, and mutation in APC impairs destruction complex formation. Over 70% of colorectal adenocarcinoma patients carry mutations in the APC gene (**Figure 3A**) that lead to the stabilization of β-catenin. APC has long been known to be an important initiator gene for the majority of colorectal cancers. However, a recent study suggests that colorectal cancer tumors with a single APC mutation can have a survival benefit, whereas tumors lacking any APC mutations convey a worse prognosis (50). Other cancers display a lower number of APC mutations, whereas endometrial carcinoma, esophagogastric adenocarcinoma, and melanoma exhibit over 10% APC mutations. An aberrant APC promoter mutation is found in early endometrial carcinoma, which decreases with cancer progression (51), suggesting that loss of APC function is an early event in endometrial carcinoma. β-catenin mutations in N-terminal serine/threonine residues or adjacent residues that interrupt CK1- and GSK3β-induced regulatory serine/threonine phosphorylations have been found in several cancers (**Figure 3B**). In endometrial carcinoma, 20-40% of patients carry mutations in β-catenin (52). It is also frequently mutated in hepatocellular carcinoma (15-33%) (53).

However, β-catenin is not mutated in pediatric T-ALL, and even if it is found to be mutated in any T-ALL cell line or patient sample, this holds no clinical significance (54). The most common activating missense mutation found in the endometroid carcinoma subtype of epithelial ovarian cancer (EOC) is in the β-catenin gene, *CTNNB1*, accounting for 54% of cases (55). This mutation occurred within the amino-terminal domain of β-catenin (55), which is positively correlated with its nuclear localization and expression of the β-catenin target genes. GSK3β phosphorylates the amino-terminal domain of β-catenin, leading to its degradation. Thus, mutations within this domain help β-catenin in evading degradation instead of accumulating in the nucleus (56). Moreover, loss-of-function mutations in genes encoding several components of the destruction complex, such as AXIN, APC, and GSK3β, were also reported in EOC, although not frequently (57). Thus, β-catenin target genes, such as *cMyc*, *CCND1*, and *VEGF*, were constitutively activated due to the disrupted WNT pathways contributed by the various mutations in its different components, which aided in cancer progression (58). 

## Non-Canonical WNT Signaling Pathway

**Figure 4:**
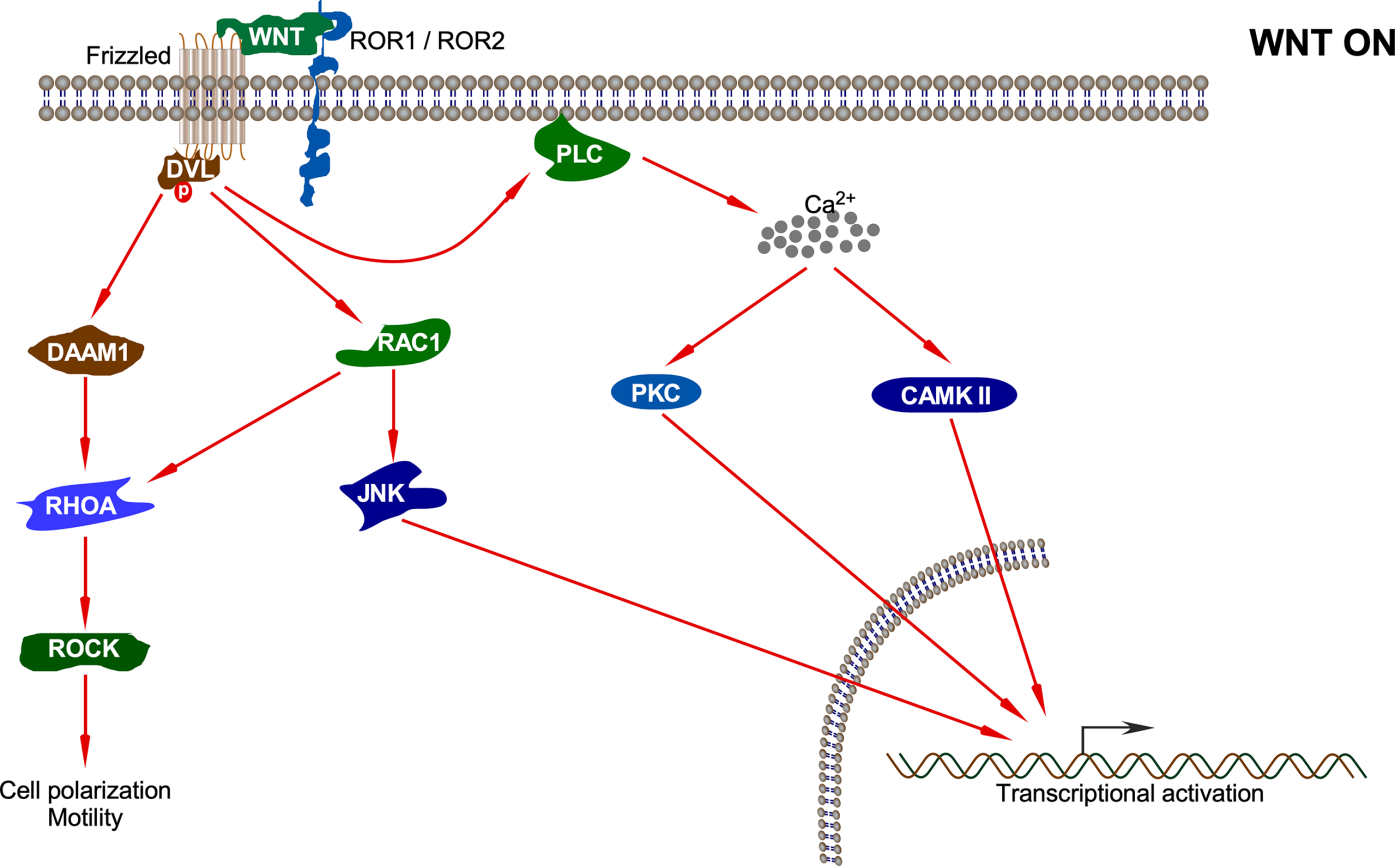
Non-canonical WNT pathway. Upon interaction of the non-canonical WNT ligand with Fz and ROR1/ROR2, several pathways including DAAM1-RHOA-ROCK, RAC1-JNK, PKC, and CAMKII pathways are activated which results in different cellular processes and transcriptional activation of different sets of genes.

Non-canonical WNT ligands such as WNT4, WNT5A, WNT5B, WNT7A, WNT7B, and WNT11 bind to Frizzled receptors (Fzd2, Fzd3, Fzd4, Fzd5, and Fzd6), and ROR1/ROR2 (receptor tyrosine kinase-like orphan receptor) or RYK acts as co-receptors to initiate non-canonical signaling (**Figure 4**). This pathway has always been defined as the one where β-catenin does not accumulate in the nucleus (59). It generally governs intercalation, cellular movement, and directed migration culminating in convergence and extension along the anterior/posterior axis of the organism (60, 61). Based on the phenotypic response, non-canonical signaling can be classified into two branches: WNT/PCP (Planar Cell Polarity) and the WNT-cGMP (cyclic guanosine monophosphate)/Ca^2+^ pathways (61, 62).

The first branch of the non-canonical pathway – PCP signaling occurs through WNT-Fz receptor interaction without the involvement of LRP5/6 co-receptors. This results in activation of the DVL protein, which in turn activates a small GTPase such as RAC (63). Activated RAC further stimulates JNK activation (64). DVL also forms a complex with DSH associated activator of morphogenesis 1 (DAAM1), which results in the activation of other GTPases: RHO and, subsequently, Rho-associated kinase (ROCK) and myosin (63). The actin remodeling process associated with cell polarization and motility is controlled by signals emanating from both RAC and RHO activation (64). Non-canonical WNT ligands such as WNT5A also interact with ROR family orphan receptor tyrosine kinases such as ROR2 to activate JNK, RHOA, etc. This has been shown to antagonize the canonical WNT signaling pathway by inhibiting the transcriptional activation potential of the β-catenin/TCF complex, thus reducing the expression of *Cyclin D1* (65, 66).

The second branch of the non-canonical pathway – WNT/Ca^2+^ signaling is characterized by WNT-Fz-induced phospholipase C (PLC) activation that increases cytoplasmic Ca^2+^ levels. Several Ca^2+^-responsive enzymes such as protein kinase C (PKC), calcineurin, and calcium/calmodulin-dependent protein kinase II (CaMKII) are activated upon sensing the intracellular Ca^2+^ flux (67). CaMKII further activates the transcription factors nuclear factor of activated T cells (NFAT), TGF-β activated kinase (TAK1), and Nemo-like kinase (NLK), all of which result in decrease in the levels of intracellular cGMP, which antagonizes the canonical WNT signaling (68, 69). Moreover, TAK1 activates NLK, which in turn phosphorylates TCF. This prevents the β-catenin-TCF complex from binding DNA, thereby inhibiting gene transcription (70). The WNT/Ca^2+^ signaling regulates various developmental processes such as cytoskeletal rearrangements, cellular adhesion, dorsoventral patterning, and tissue separation in embryos (71).

The non-canonical WNT signaling pathway also has the potential to inhibit canonical WNT signaling by promoting the proteasomal degradation of β-catenin through an alternative E3 ubiquitin ligase complex containing APC, Ebi, and Siah1 or Siah2 (72, 73). This does not involve GSK3β or β-TrCP and neither requires activation of CaMKII or NFAT (74). Thus, the antagonism of canonical WNT signaling by non-canonical WNTs involves multiple mechanisms that may or may not involve calcium (71).

## Coactivators Regulating the Output of the WNT Signaling Pathways

The canonical WNT signaling pathway is dependent on β-catenin, whereas the non-canonical WNT signaling pathway is independent of β-catenin (75). Once in the nucleus, β-catenin interacts with members of the TCF/LEF family of transcription factors. β-catenin can also interact with a variety of other transcription factors like FOXO, HIF1, Sox family members, nuclear receptors, etc. Thus, nuclear β-catenin can perform divergent functions, which adds complexity to the interpretation of canonical WNT signaling (76). Transcriptional coactivators like cAMP response element-binding protein (CREB)-binding protein (CBP) or its closely related homolog, p300, are key regulators of RNA polymerase II-mediated transcription (77). β-catenin can recruit either CBP or p300 along with other components of the basal transcriptional machinery to generate a transcriptionally active complex, which leads to the expression of a variety of downstream target genes (78). Differential utilization of these coactivators by β-catenin can lead to different cellular outputs (75). Canonical WNT signaling pathway mediated by WNT3A stabilizes β-catenin and leads to its association with the coactivator CBP for transcribing genes related to self-renewal, potency, and proliferation (77). However, the non-canonical WNT signaling pathway mediated by WNT5A induces the activation of various kinases such as PKC, CaMKII, SIK, AMPK, and MAPK (5, 8, 79). PKC further phosphorylates Ser89 of p300, which increases its affinity for β-catenin (77). The association of coactivator p300 with β-catenin drives the gene expression program from a proliferative state to a differentiative state; that also governs planar cell polarity, convergent extension, and cytoskeletal reorganization (80). Thus, coordinated integration between both canonical and non-canonical WNT signaling pathways is extremely crucial for regulating cell proliferation with differentiation and adhesion (75). Canonical and non-canonical WNT signaling pathways are, hence, highly dynamic, coupled, and not mutually exclusive with cross-talk occurring between them, which is dependent on the type of cell, tissue, and specific stage of development (77). 

## Kinases Regulating the Activity of β-Catenin

Alternative signal transduction pathways from various growth factor receptors and ion channels can activate a myriad of kinases, which also have the potential to phosphorylate either CBP or p300, thereby controlling differential utilization of coactivators by β-catenin. Moreover, these kinases can phosphorylate β-catenin directly (77), thereby playing key roles in regulating the localization, expression, and function of β-catenin (81). We will be discussing some of those kinases in the following sections.

### Cell Cycle-Associated Kinases

Aurora kinase A (AURKA) is required for centrosome function and spindle assembly during mitosis that, once activated, phosphorylates Polo-like kinase 1 (PLK1) at Thr210 in presence of the co-factor Bora (82–85). PLK1 is the master regulator of the cell cycle, playing an important role in M-phase progression (86). PLK1 in turn phosphorylates several substrates, FOXM1 being one of them. FOXM1 then transcribes target genes involved in cell cycle progression, cell proliferation, genomic stability, chemoresistance, and DNA damage repair (87–89). All components of the AURKA-PLK1-FOXM1 axis appear to be hyperactivated due to multiple events associated with a BCR-ABL fusion protein that promotes resistance to tyrosine kinase inhibitors in chronic myeloid leukemia (CML) (90–94). The AURKA-PLK1-FOXM1 axis interacts with β-catenin, which supports the resistance of BCR-ABL+ leukemic stem cells to tyrosine kinase inhibitors (86, 95–97). PLK1 physically interacts with β-catenin and phosphorylates it on Ser718 in the M phase of the cell cycle, indicating an important M-phase specific function of β-catenin such as the regulation of centrosomes (86). Deregulated PLK1 expression has been reported in many cancers, which in turn could alter β-catenin regulation in the M phase, thus leading to abnormal cell cycle control and chromosome instability (79, 86, 98). Moreover, PLK1 regulates the activity of another cell cycle regulatory kinase, NIMA-related protein kinase 2 (Nek2). Nek2 phosphorylates Thr41, Ser37, and Ser33 at the N-terminus of β-catenin along with some five additional sites; these are the amino acid residues phosphorylated by GSK3β as well. This inhibits the interaction of β-catenin with β-TRCP, thereby stabilizing it. Nek2 regulates centrosome disjunction/splitting; thus, β-catenin stabilized by Nek2 accumulates at centrosomes in mitosis, regulating the centrosome cycle (81). AURKA sequesters AXIN from the destruction complex, while FOXM1 associates with β-catenin, thereby enabling its nuclear import and recruitment to the TCF/LEF transcription complex to support leukemic cell proliferation and survival (99). So, all 3 components of the AURKA-PLK1-FOXM1 axis regulate β-catenin transcriptional activity. Inhibition of these components ultimately results in the dephosphorylation of FOXM1, causing the release of β-catenin from its binding, which leads to its cytoplasmic relocation and degradation. The proliferation and survival of BCR-ABL+ cells are thus blocked by the inhibition of the β-catenin-mediated transcriptional activity (100).

### Protein Kinase C (PKC)

Once activated, β-catenin is translocated to the nucleus. Its stability is regulated by the E3 ubiquitin ligase tripartite motif-containing protein 33 (TRIM33), which is independent of GSK3β and β-TRCP (101). However, this regulation is dependent on the PKC family member PKCδ, which is activated upon prolonged WNT stimulation. PKCδ phosphorylates β-catenin at Ser715, thereby facilitating its interaction with TRIM33. β-catenin is then targeted for degradation, shutting off the WNT pathway. Apart from inhibiting the canonical WNT pathway mediated by WNT3a, TRIM33 can inhibit EGF-induced β-catenin transactivation (101). TRIM33 is thus known to act as a tumor suppressor in various cancers including clear cell renal cell carcinoma (102), chronic myelomonocytic leukemia (103), hepatocellular carcinoma (104), and pancreatic cancer (105). In fact, PKC is a family of 10 protein serine/threonine kinases that are encoded by 9 genes (106–109). PKC family members are divided into three subfamilies: classical (PKCα, PKCβ1, PKCβ2, and PKCγ), novel (PKCδ, PKCϵ, PKCη, and PKCθ) and atypical (PKCζ and PKCι) (108, 109). Classical and novel PKC isoforms display dependency on second messengers for activation. For example, classical PKC isoforms are diacylglycerol (DAG) and Ca^2+^-responsive while novel PKC isoforms are dependent on DAG. While several PKC family members have been implicated in tumorigenesis, PKCδ acts as a tumor suppressor, as its main function is to induce apoptosis, apart from regulating β-catenin degradation (101, 108–114). Another PKC family member that serves as a tumor suppressor in intestinal cancer is the atypical PKCζ that induces Ser45 phosphorylation of β-catenin, which is independent of CK1α and is thus important for GSK3β-mediated phosphorylation (115). The classical PKC isoform PKCα induces phosphorylation of β-catenin at N-terminal serine residues, which in turn results in enhanced proteasomal degradation of β-catenin and a reduction of transcriptional regulations (116). Furthermore, PKCα phosphorylates ROR1 (RORα) at Ser35, which can eventually limit the transcriptional regulation of β-catenin (117). Collectively, these studies suggest that several PKC isoforms play important roles in the regulation of WNT/β-catenin signaling.

### Protein Kinase A (PKA)

The cyclic AMP (cAMP)-dependent protein kinase, protein kinase A (PKA), has diverse cellular functions including cell proliferation, differentiation, cell cycle regulation, and apoptosis. The cAMP/PKA pathway plays a highly complex and cell-specific role in regulating cell growth, as it stimulates growth for some cell types while inhibiting others (118, 119). PKA can even have contrasting effects of promoting or inhibiting cell proliferation in the same cell type, such as the vascular smooth muscle cells, depending on the agonist that stimulates its activity (120, 121). The presenilin1 complex in Alzheimer’s disease contains presenilin1, GSK3β, the catalytic subunit of PKA and β-catenin. PKA induces Ser45 phosphorylation on β-catenin, thereby enhancing GSK3β-dependent phosphorylation of β-catenin and its subsequent proteasomal degradation, which is independent of the WNT-controlled AXIN complex (122). However, in contrast, activation of PKA has also been shown to increase β-catenin accumulation in both the cytosol and nucleus of HEK293 cells after stimulation with prostaglandin E2 (PGE2) (123). PKA phosphorylates β-catenin on Ser552 and Ser675 that stabilize β-catenin by inhibiting its ubiquitination without affecting the formation of the destruction complex and GSK3β-dependent phosphorylation (123–125). PKA-induced phosphorylation of β-catenin at Ser675 promotes TCF/LEF transactivation and binding to its transcriptional coactivator CREB-binding protein (CBP) (124). Thus, the phosphorylation of β-catenin by PKA at different serine residues determines the outcome of β-catenin regulation. 

### Receptor Tyrosine Kinases (RTKs)

Receptor tyrosine kinase (RTK) consists of a family of around 60 mammalian protein tyrosine kinases (106, 107). The RTK epidermal growth factor receptor (EGFR) activates AKT that directly phosphorylates β-catenin on Ser552, which increases the cytosolic and nuclear β-catenin levels and transcriptional regulation, thereby promoting tumor cell invasion (126). Furthermore, the proliferation of PTEN-deficient intestinal stem cells that initiate intestinal polyposis is driven by AKT activation where AKT phosphorylates β-catenin on Ser552 (127). Ultraviolet (UV) irradiation activates EGFR in keratinocytes, resulting in the phosphorylation of β-catenin at Tyr654, which is responsible for its dissociation from the E-cadherin/β-catenin/α-catenin complex (128). Furthermore, UV-induced EGFR activation allows for the nuclear translocation of β-catenin and transcriptional activation through interaction with TCF4 (128). Fibroblast growth factor-2 (FGF-2) activates MAP kinase signaling in osteoblasts, which in turn phosphorylates β-catenin. The phosphorylation is mediated by MEKK2 (MAP3K2) at Ser675 that stabilizes β-catenin and increases its activity by recruiting the deubiquitinase USP15 (129). In colorectal cancer, a loss-of-function mutation in APC is very common, stabilizing β-catenin due to the lack of destruction complex. However, 60% of colorectal cancer patients carry mutations in KRAS and BRAF genes that result in uncontrolled MAPK signaling. Oncogenic activation of KRAS/BRAF/MEK signaling increases the transcriptional activities of β-catenin/TCF4 and c-MYC promoter and increases the mRNA levels of c-Myc, AXIN2, and Lef1 (130). Another MAPK, p38γ, phosphorylates β-catenin at Ser605 (131). FGFR2, FGFR3, EGFR, and TRKA increase the cytosolic β-catenin concentration by dissociating β-catenin from cadherin complex through direct phosphorylation at Tyr142 (132). The RTK MET interacts with β-catenin in hepatocytes, colon cancer, and breast cancer cell lines; this association occurs at the region of cell-cell contact (133, 134). The association is constitutive but can be abrogated by hepatocyte growth factor (HGF) stimulation. HGF induces tyrosine phosphorylation of β-catenin at Tyr654 and Tyr670, resulting in its dissociation from MET (135). Hepatocyte growth factor-like protein (HGFL) induced RON activation, which resulted in tyrosine phosphorylation of β-catenin at Tyr654 and Tyr670, inducing its nuclear accumulation and transcriptional activation in breast cancer (136). Type-1 insulin-like growth factor (IGF-1) causes the nuclear translocation of β-catenin in the context of IGF-1R signaling, which leads to the activation of β-catenin target genes such as *c-MYC* and *cyclin* (137–141). This is brought about by the direct binding of sequences between amino acid residues 695 and 781 in the C-terminus of β-catenin to sequences located between amino acid residue 600 and the C-terminus of insulin receptor substrate-1 (IRS-1), in both the nucleus and the cytoplasm (142). The PTB domain of IRS-1 then translocates the β-catenin-IRS-1 complex to the nucleus (143). β-catenin binding to IRS-1 with its C-terminus may thus prevent phosphorylation at its N-terminus by GSK3β, which primes β-catenin for ubiquitination and degradation (8, 144). So, IRS-1, a docking protein for both IGF-1 and insulin receptors, can regulate the subcellular localization and activity of β-catenin in cells that are responsive to the mitogenic action of IGF-1 (142, 145).

### Janus Kinase 3 (JAK3)

Adherens junctions are multiprotein complexes in cell-cell junctions that connect neighboring cells to maintain the epithelial tissue structure (146). β-catenin is an adherens junctions-associated protein that links the cytoplasmic domain of cadherins to the α-catenin-associated actin cytoskeleton and has been implicated in adherens junctions remodeling (16). Janus kinase 3 (JAK3) is a non-receptor tyrosine kinase that transmits intracellular signals through interactions with the *γ* chain of several cytokine receptors upon stimulation with cytokines (147). It associates with β-catenin in adherens junctions and phosphorylates Tyr30, Tyr64, and Tyr86 (148). However, JAK3-mediated phosphorylation is required prior to the Tyr654 phosphorylation of β-catenin. Phosphorylation on those sites by JAK3 suppressed EGF-induced epithelial-mesenchymal transition (EMT) and, rather, induced epithelial barrier functions by adherens junctions localization of phosphorylated β-catenin *via* its association with α-catenin. Moreover, a reverse effect was seen with increased EMT and compromised epithelial barrier functions upon the loss of JAK3-mediated β-catenin phosphorylation, which even abrogated the localization of β-catenin in the adherens junctions (148).

### p21-Activated Kinase (PAK)

p21-activated kinase (PAK) is a family of six protein serine/threonine kinases that acts mainly as the effector proteins for the Rho GTPases CDC42 and RAC (149). PAK-family proteins regulate various cellular processes including cell proliferation and cell survival, cell motility, cytoskeletal reorganization, oncogenic transformation, and gene transcription (149, 150). JNK2 and PAK1 both are the downstream effectors of RAC1. More advanced colon cancer samples often contained K-Ras mutation, which elevated two signaling branches: K-Ras/RAC1/JNK2 cascade and K-Ras/RAC1/PAK1 cascade (151–154). Thus, JNK2-mediated phosphorylation of β-catenin at Ser191 and Ser605 increased, which led to its nuclear translocation (151, 152). Additionally, RAC1 induces β-catenin Ser191 and Ser605 phosphorylation, which is mediated by JNK2; phosphorylation on those sites is required for the nuclear localization of β-catenin (151, 155). Apart from RAC1, the EGF and IGF signaling pathways may induce PAK1 expression (156–158). Another kinase, PAK4, shuttles between the nucleus and cytoplasm and interacts with β-catenin in both cellular compartments (159). It was observed that both PAK1 and PAK4 mediated phosphorylation of β-catenin on Ser675 stabilizes it by inhibiting its degradation and thus promotes TCF/LEF transcriptional activity (159, 160). Therefore, one downstream effector of RAC1 mediates the nuclear translocation of β-catenin, while the other downstream effector promotes its transcriptional activity. 

### Tyrosine Kinases

Several tyrosine phosphorylation sites have been identified on β-catenin and have been shown to play important roles. In the presence of a disrupted cellular contact and active TGF-β1, E-cadherin and the epithelial integrin α3β1 associate with TGF-β1 receptors, where Tyr654 in β-catenin gets phosphorylated by the epithelial integrin in primary alveolar epithelial cells (AECs) and the phosphorylated β-catenin forms a complex with phospho-SMAD2, resulting in EMT initiation in idiopathic pulmonary fibrosis (IPF) (161, 162). This epithelial integrin-mediated cross-talk between WNT and TGF-β1 signaling pathways, which is required for pulmonary fibrogenesis and EMT, is done with the help of TGF-β1mediated activation of SRC family kinases (163). In a hypoxic condition, reactive oxygen species (ROS) activates SRC that phosphorylates β-catenin on Tyr654. This tyrosine-phosphorylated β-catenin gets complexed with SRC and HIF1α in primary human lung adenocarcinomas and lung tumor cell lines to promote the transcriptional activity of HIF1α and hypoxia-induced EMT (164). Another substrate identified for SRC in β-catenin is Tyr86 (165). An SFK FYN induces tyrosine phosphorylation of β-catenin at Tyr142 and disrupts its association with α-catenin (37). Other tyrosine kinases such as FER, FES, and c-MET also participate in the phosphorylation of Tyr142 (37). This disrupts the binding of α-catenin to β-catenin and, instead, favors the binding of β-catenin to the nuclear transporter B-cell lymphoma 9 (Bcl9), which acts as a co-activator in WNT signaling (166). Protein tyrosine kinase (PTK6) is a distant SRC family member. It is regulated by C-terminal tyrosine phosphorylation, which is similar to SRC but lacks an N-terminal myristoylation site and, therefore, cannot localize to the membrane-like other SRCs. PTK6 interacts with both the cytoplasmic and nuclear β-catenin and directly phosphorylates β-catenin at several tyrosine residues including Tyr64, Tyr142, Tyr331, and Tyr333 (167). In the nucleus, PTK6 inhibits β-catenin function, which was shown to be independent of tyrosine phosphorylation, suggesting a possible kinase-independent role of PTK6 in the transcriptional regulation of β-catenin function (167). 

### BCR-ABL Fusion Protein

The BCR-ABL fusion protein, which is frequently found in chronic myeloid leukemia (CML), physically interacts with β-catenin and phosphorylates it at Tyr86 and Tyr654 (168). Phosphorylation of these tyrosine sites by BCR-ABL prevents the association of β-catenin with AXIN/GSK3β complex; thereby, serine/threonine phosphorylation is prevented. This subsequently increases the cytosolic and nuclear accumulation of β-catenin that is required for self-renewal of BCR-ABL-positive CML cells (169). This process can be reversed by tyrosine phosphatase SHP1, where SHP1-mediated dephosphorylation of Tyr86 and Tyr654 restores GSK3β-dependent serine/threonine phosphorylation and, thereby, β-catenin degradation (170). In another mechanism, BCR-ABL tyrosine kinase controls β-catenin mediated transcriptional activity indirectly, without physical interaction. Chibby1 (CBY1) is a small protein that represses β-catenin transcriptional activation; it interacts with the C-terminal activation domain of β-catenin that hinders its binding with the TCF/LEF transcription factors (171). The scaffolding protein 14-3-3 drives the nuclear export of CBY1 and β-catenin by forming a stable tripartite complex with them (172, 173). However, BCR-ABL fusion protein downregulates CBY1 at the transcriptional level by promoter hypermethylation and at the post-transcriptional level by directing CBY1 toward proteasome-dependent degradation through SUMOylation (171–173). This retains β-catenin in the nucleus and sustains its activation, which is required for the proliferation of CML cells (174). 

### Casein Kinase 2 (CK2)

Casein kinase 2 (CK2) is ubiquitously expressed in the nucleus and cytoplasm of eukaryotic cells (175). CK2 phosphorylates many transcription factors, tumor suppressors, and proto-oncoproteins involved in cancer, thereby regulating a multitude of cellular processes. One such important function is the regulation of protein stability, thus contributing to cell proliferation and transformation (176). It also plays an important role in embryonic development. CK2 phosphorylates β-catenin at Thr393, which leads to its stabilization and increases its contributions in transcriptional regulations (176). This is probably mediated by decreased affinity to AXIN in the destruction complex (177). Another study identified Ser29, Thr102, and Thr112 in β-catenin as CK2 phosphorylation sites that were required for interaction with α-catenin. CK2 phosphorylation of these residues was also important for degradation of β-catenin, as pre-phosphorylation of β-catenin by CK2 stabilizes its binding to components of the destruction complex, AXIN, and GSK3β, further enhancing the activity of GSK3β. Thus, the cytoplasmic turnover of β-catenin is controlled by the combined action of CK2 and GSK3β (178). Therefore, CK2 appears to have dual roles in β-catenin regulation: one for canonical WNT signaling and the other for cell adhesion (166).

### Protein Kinase D1 (PKD1)

Protein kinase D1 (PKD1) lies downstream of the signaling pathways initiated by diacylglycerol and PKC. Diacylglycerol dictates the intracellular localization of PKD1, while PKC activates it by phosphorylation (179). PKD1 served as a tumor suppressor in advanced prostate cancer, where its expression was downregulated; later, it was found that it interacted with E-cadherin (180, 181). PKD1 interacts with β-catenin and phosphorylates it on Thr112 and Thr120 that inhibit the nuclear localization of β-catenin, thereby decreasing its transcriptional activity. This was probably because these threonine phosphorylations on β-catenin increased its interaction with α-catenin and E-cadherin, thereby linking the cytoskeleton. This suggests that Thr120 phosphorylation is critical for cell-cell adhesion (182, 183). However, it has been suggested that PKD1 expression is transcriptionally repressed by β-catenin, indicating the involvement of a negative auto-regulatory loop (184).

**Figure 5:**
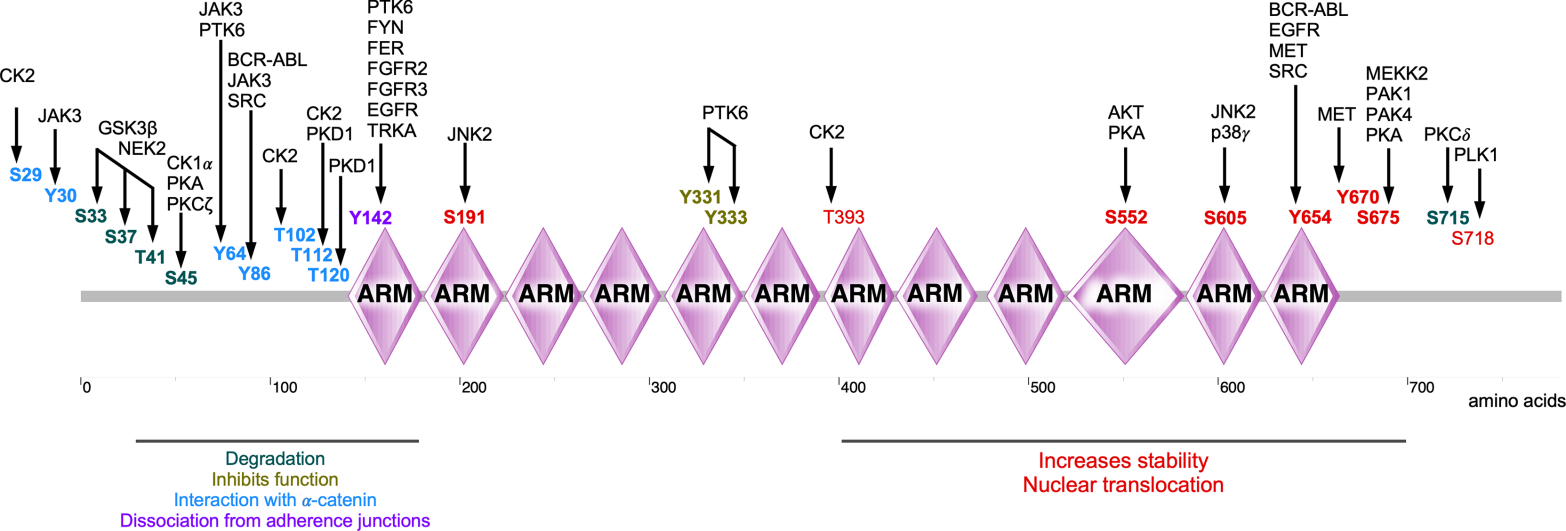
Phosphorylations sites of β-catenin. Several kinases are involved in the phosphorylation of β-catenin, thereby regulating its stability, localization, and activity of β-catenin.

In summary, β-catenin activity is tightly controlled by a series of tyrosine and serine/threonine kinases (summarized in **Table 1**). Phosphorylation on a specific β-catenin residue or several residues can affect its stability, localization, and interaction with other partners (**Figure 5**). For example, phosphorylation by tyrosine or serine/threonine kinases in the N-terminal region (except for Tyr86 and Ser191) tags β-catenin for degradation or inhibits its nuclear translocation. On the other hand, phosphorylation of the C-terminal region increases the stability of β-catenin as well as its nuclear translocation (except for Ser718). Nevertheless, different regions of β-catenin display affinity to different regulatory proteins probably due to phosphorylation modifications (8). Since a deregulated β-catenin function can contribute to malignancy, kinases involved in the regulation of β-catenin stability, localization, and function can be attractive targets for therapeutic implications. Pharmacological inhibitors against BCR-ABL, EGFR, and MET have been developed for several cancers (185–187) and probably can be repurposed to regulate β-catenin activity. However, inhibition of kinases that promote β-catenin degradation or inhibits its function can result in transcriptional activation of WNT/β-catenin target genes. 

**Table 1:** Kinases regulating β-catenin activity.

Sites	Kinases	Function	Reference
S29, T102	CK2	Degradation, interaction with α-catenin.	(176, 178)
Y30	JAK3	Interaction with α-catenin.	(148)
S33, S37, T41	GSK3β, NEK2	Degradation, contributes to mitosis.	(24, 36, 38, 81)
S45	CK1α, PKA, PKCζ	Degradation, regulation of phosphorylation.	(36, 115, 122, 123)
Y64	JAK3, PTK6	Inhibits function in the nucleus, interaction with α-catenin.	(148, 167)
Y86	BCR-ABL, JAK3, SRC	Increases the cytosolic and nuclear accumulation, interaction with α-catenin.	(148, 165, 168, 169)
T112	CK2, PKD1	Degradation, interaction with α-catenin, inhibits the nuclear localization.	(178, 182)
T120	PKD1	Interaction with α-catenin, inhibits the nuclear localization	(182, 183)
Y142	PTK6, FYN, FER, FGFR2, FGFR3, EGFR, TRKA	Inhibits function in the nucleus, dissociation from adherence junctions, cytoplasmic accumulation.	(25, 35, 37, 132, 167)
S191	JNK2	Nuclear translocation.	(151, 152)
Y331, Y333	PTK6	Inhibits function in nucleus.	(167)
T393	CK2	Stabilization, enhances contributions in transcriptional regulations	(176)
S552	PKA, AKT	Inhibits ubiquitination, stabilization, transcriptional regulation, and promoting tumor cell invasion.	(124, 126, 127)
S605	JNK2, p38γ	Nuclear translocation.	(131, 151, 152)
Y654	MET, EGFR, SRC, BCR-ABL	Dissociation from the E-cadherin/β-catenin/α-catenin complex, nuclear translocation.	(128, 135, 164, 168, 169)
Y670	MET	Nuclear translocation.	(135)
S675	PAK1, PAK4, MEKK2, PKA	Stabilization, inhibits ubiquitination, contributes to TCF/LEF transactivation.	(123, 124, 129, 159, 160)
S715	PKCδ	Facilitate interaction with TRIM33 and degradation.	(101)
S718	PLK1	Regulation of centrosomes in M phase	(86)

## Conclusion

Like other cellular signaling events, WNT/β-catenin signaling is tightly controlled by protein phosphorylation and dephosphorylation. For example, in classical WNT/β-catenin signaling, the formation and activity of the destruction complex are phosphorylation-dependent, which further regulates the stability of β-catenin in a phosphorylation-dependent manner (8). Nevertheless, as discussed above, several kinases are involved in the regulation of this evolutionarily conserved pathway modulating cellular adhesion, polarity, motility, migration, proliferation, and differentiation (188–190).

Much is already known about WNT/β-catenin signaling and the roles of its components in various cancers. However, there are still several possible missing links that need to be studied in the regulation of WNT/β-catenin. For instance, aurora family kinases seem to be involved in the stabilization of β-catenin (191). However, the exact mechanism of how β-catenin stabilization is mediated by aurora family kinases remains to be determined. Although WNT/β-catenin signaling is highly simplified in the figures depicted in this review, the interaction between various signaling proteins as described above makes it highly complicated. Both the canonical and non-canonical pathways involving β-catenin intersect at many levels, which adds several layers of complexity. Thus, attempts should be made to precisely define and dissect these pathways, so that they can be better targeted as therapies in the future.

## Author Contributions

KS and JK outlined the content, reviewed the literature, and wrote the manuscript. All authors contributed to the article and approved the submitted version. 

## Funding

This research was supported by the Kungliga Fysiografiska Sällskapet i Lund (KS), the Crafoord Foundation (JK), Magnus Bergvalls Stiftelse (JK), the Swedish Cancer Society (JK), and the Swedish Childhood Cancer Foundation (JK).

## Conflict of Interest

The authors declare that the research was conducted in the absence of any commercial or financial relationships that could be construed as a potential conflict of interest.

## Publisher’s Note

All claims expressed in this article are solely those of the authors and do not necessarily represent those of their affiliated organizations, or those of the publisher, the editors and the reviewers. Any product that may be evaluated in this article, or claim that may be made by its manufacturer, is not guaranteed or endorsed by the publisher.
